# Managing Severe Asthma: A Role for the Long-Acting Muscarinic Antagonist Tiotropium

**DOI:** 10.1155/2018/7473690

**Published:** 2018-10-24

**Authors:** Eckard Hamelmann

**Affiliations:** Children's Center Bethel, Ev. Klinikum Bielefeld gGmbH, Grenzweg 10, 33617 Bielefeld, Germany

## Abstract

Severe asthma is associated with substantial morbidity and mortality. Therapies must be maximized to gain control of a patient's severe asthma; however, avoiding overtreatment is also important. The mainstays of asthma maintenance treatment are inhaled corticosteroids (ICS) and long-acting *β*_2_-agonsits (LABAs), with the option of supplementary add-on treatments. New add-on treatments for severe asthma have emerged over the past two decades, including personalized biological therapies that are guided by a patient's asthma phenotype. In addition, the long-acting muscarinic antagonist tiotropium has been recommended as an add-on treatment for severe asthma. Phase III clinical trials have shown tiotropium in combination with ICS/LABA to be efficacious in patients with severe asthma. Further analyses of clinical trial data have indicated that there is no benefit in stratifying patients by phenotype to predict tiotropium efficacy. Furthermore, health economic studies suggest tiotropium to be a cost-effective treatment in patients with severe asthma. This review will present the evidence surrounding the role of tiotropium in severe asthma and will discuss the use of tiotropium add-on therapy before personalized medicine strategies in the stepwise process of gaining asthma control.

## 1. Introduction

For the estimated 358 million patients worldwide who live with asthma, management of their disease has the overarching goal of gaining complete control and minimizing future risk [1]. Control is defined as the suppression of asthma symptoms and exacerbations, the removal of rescue medication need, restoration of normal lung function, and the reversal of activity limitation due to asthma [2]. Moreover, control of asthma includes reductions in the future risk of exacerbations, lung function decline, worsening control, and medication increase. In fact, current control has been shown to predict future risk of exacerbations, instability, and future lung function decline [3, 4]. However, asthma severity varies greatly between patients [5]. Accordingly, recommended treatment strategies also vary, with more aggressive treatment recommended for more severe asthma in order to gain control of the disease. Furthermore, the aim is for the patient to achieve asthma control whilst experiencing minimal treatment side effects [5]. This means patients should receive only the therapy required to achieve complete control and not unnecessary additional interventions.

Despite treatment in accordance with guidelines, including the use of inhaled corticosteroids (ICS) and/or long-acting *β*_2_-agonists (LABA), a proportion of patients continue to have impaired control and experience the symptoms of asthma [2, 5, 6]. For these uncontrolled patients, treatment may be increased in the form of dosage or employing additional therapies [5]. Extrinsic factors such as low adherence to therapy, a reluctance of patients and carers to use corticosteroids, insufficient patient and clinician disease education, comorbidities, and environmental risk factors (for example, allergens and tobacco smoke) also contribute to uncontrolled asthma [5, 7–9]. Poor management of these extrinsic factors defines difficult-to-treat asthma [5].

**Figure 1:**
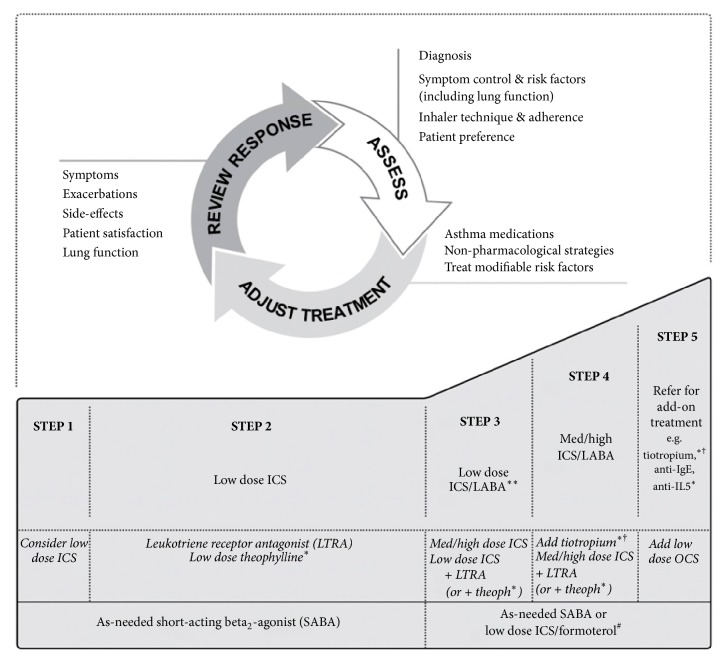
Stepwise asthma management in adults, adolescents, and children aged 6–11 years.* Notes*. ^*∗*^Not for children aged 12 years; ^*∗∗*^for children aged 6–11 years (preferred Step 3 treatment medium-dose ICS); ^#^for patients prescribed BDP/formoterol or BUD/formoterol maintenance and reliever therapy; ^†^tiotropium by mist inhaler is an add-on treatment for patients aged ≥12 years with a history of exacerbations. Copyright ©2018 Global Initiative for Asthma. Reproduced with permission from. Global Initiative for Asthma. Global strategy for asthma management and prevention. 2018.* Abbreviations*. BDP, beclomethasone dipropionate; BUD, budesonide; ICS, inhaled corticosteroid; LABA, long-acting *β*_2_-agonist.

The Global Initiative for Asthma (GINA) 2018 asthma management strategy follows a stepwise escalation in therapy so as to gain control of a patient's asthma (Figure 1) [5]. GINA 2018 defines severe asthma as asthma that remains uncontrolled despite, or that is only controlled by, Steps 4–5 of treatment; these steps are comprised of two or more controllers, usually medium-to-high dose ICS/LABA, plus as-needed reliever medication (Figure 1, Steps 4–5) [5]. The morbidity and mortality of patients with severe asthma are substantial: 26% of patients are not working due to their disease, and an estimated 39% of asthma deaths are of patients with severe asthma [10, 11]. Severe refractory asthma—a subset of severe asthma cases, defined as uncontrolled asthma despite management of extrinsic factors—represents an estimated 3.6% of asthma cases, equating to 12.9 million cases worldwide [1, 6].

Over the past two decades, the available spectrum of add-on drugs approved for use in asthma has broadened. These include small-molecule leukotriene modifiers and monoclonal antibodies, both of which target the immune component of asthma, as well as bronchodilators [12]. In addition to the development of new drugs, research into the pathology of asthma has revealed the disease to be a complex and heterogeneous disease. Patients can now be stratified into different subtypes of asthma, such as allergic or type 2-high (T2-high) phenotypes [13]. This involves the measurement of biomarkers such as blood eosinophil count, blood immunoglobulin E (IgE) levels, and the fraction of exhaled nitric oxide [13]. Personalized therapy plans can then be tailored to each patient in accordance with their subtype of the disease (see other reviews in this special issue). Clinical guidelines reflect these developments, with the GINA 2018 report suggesting patients with severe asthma who remain uncontrolled on ICS/LABA may be phenotyped and treated with appropriate biological therapies [5]. However, phenotyping patients may be time-consuming, and phenotypes may not be stable over time [14, 15]. Furthermore, personalized therapies are expensive, primarily constituting monoclonal antibody-based drugs, and are not widely available for patients under the age of 18.

Long-acting muscarinic antagonists (LAMAs) are a class of bronchodilators with a mechanism of action that is distinct from LABAs. Inhibition of the muscarinic receptors of the bronchioles causes relaxation of the smooth muscle; furthermore, inhibition has been shown to reduce inflammation and asthma-related airways remodelling in preclinical asthma models [16–19]. Tiotropium is the first LAMA add-on therapy approved for use in asthma. This review will present the evidence surrounding the role of tiotropium add-on therapy in severe asthma management and discuss how it may be a broadly effective and economical therapy for use before personalized medicine strategies.

## 2. Where Do LAMAs Fit into Severe Asthma Management?

As described in the GINA 2018 report, achieving asthma control requires a cyclical approach to patient management (Figure 1) [5]. Patients are initially assessed for asthma control: if their disease is uncontrolled, new treatment may be provided; if the patient has had 3 months of asthma control, a reduction in treatment may be considered [5]. Reviewing the impact of changes in treatment on asthma control allows patients and clinicians to make a judgement on whether treatment should be adjusted, thereby restarting the assessment cycle. However, this process relies upon the clinician and the patient ensuring all symptoms are accurately reported and assessed, appropriate treatments are trialled, and treatments are properly adhered to. In fact, an estimated 79.5% of uncontrolled asthma cases are thought to be due to failure to adhere to asthma medications and poor inhaler technique, rather than truly medication-resistant disease [6].

Tiotropium is a new addition to the range of treatments that may be trialled in asthma patients experiencing suboptimal asthma control. First approved for use in asthma in 2014, tiotropium is licenced for use as a once-daily maintenance add-on therapy in patients aged 6 years and older in the US and EU and in patients aged 15 years and older in Japan [20–22]. GINA recommends tiotropium for use in severe asthma (Steps 4 and 5) as an add-on treatment to medium-to-high dose ICS/LABA in patients aged ≥12 years (Figure 1) [5]. Specifically, GINA placed tiotropium beginning with Step 4 treatment and before biologics or oral corticosteroids (OCS) (Figure 1). Similarly, German, Spanish, and UK asthma guidelines recommend tiotropium add-on use in patients with severe asthma as an option for add-on therapy when high-dose ICS/LABA therapies fail to gain asthma control; however, this recommendation is for adults only [2, 23, 24].

### 2.1. Clinical Studies Investigating Tiotropium in Patients with Severe Asthma

Current guidelines have based their recommendations on evidence from Phase III clinical studies investigating the use of tiotropium add-on therapy in severe asthma (Table 1). In the two replicate Phase III PrimoTinA-asthma trials, 912 adult patients with symptomatic severe asthma received either tiotropium 5 *μ*g or placebo, delivered by the Respimat Soft Mist inhaler, as add-on maintenance therapy to at least ICS/LABA [25]. The first co-primary endpoint—change from baseline (response) in peak forced expiratory volume in 1 second (FEV_1_) within 3 hours after dose (FEV_1(0–3h)_) at Week 24—was significantly greater in patients receiving tiotropium add-on compared with placebo (86–154 mL, P<0.05). The second co-primary endpoint—trough FEV_1_ response at Week 24—was significantly greater in the tiotropium add-on arm compared with the placebo arm (88–111 mL, P<0.05). The third co-primary endpoint—the time to the first severe asthma exacerbation (an exacerbation was defined as deterioration of asthma requiring OCS for ≥3 days)—was increased with tiotropium by 56 days compared with placebo (282 days versus 226 days). This corresponded to a reduction in risk of exacerbation of 21% with tiotropium compared with placebo (odds ratio [OR] 0.79, P=0.03), with the total number of exacerbations per patient-year being 0.53 and 0.66 for patients receiving tiotropium or placebo, respectively. This result shows that tiotropium can reduce the number of patients with severe asthma requiring OCS. This effect was despite inclusion criteria for the trials where patients were only required to have had a minimum of one exacerbation in the past year. Therefore, in contrast to recent trials for biologics [26–29], patients with a subtype of asthma that was highly prone to exacerbation were not specifically selected. Nonetheless, an increased median time to first asthma worsening—a secondary endpoint defined as either a progressive increase in symptoms or a decline of ≥30% in morning peak expiratory flow at screening for 2 consecutive days—was also found (hazard ratio 0.69, P<0.001). In line with this, the PrimoTinA-asthma trials showed some indication that tiotropium provides improvements in asthma symptom control, a secondary endpoint for the trials. Trial 2 of PrimoTinA-asthma showed a significant improvement in patients' seven-question Asthma Control Questionnaire (ACQ-7) score (–0.2, P=0.003), even though the effect in trial 1 did not reach statistical significance (–0.13, P=0.06). In a post hoc pooled analysis of both trials, ACQ-7 responder rate (a responder was defined by having a decrease in ACQ-7 score from baseline ≥0.5, which is considered the minimum clinically important difference) was significantly improved at Week 24 (OR 1.32, P=0.04) and at Week 48 (OR 1.68, P<0.001) [30]. Taken together, the lung function improvements, exacerbation and asthma worsening reductions, and symptom reductions reported in the PrimoTinA-asthma trials show that tiotropium has utility in gaining asthma control for adult patients with severe asthma.

Efficacious add-on therapies for paediatric patients with severe asthma are of particular interest as they may reduce the need to increase ICS dose, which is associated with a reduction in growth [31, 32]. A Phase III trial in adult patients has shown tiotropium to be superior to doubling ICS dose in terms of the proportion of days with asthma control, improvement in lung function, and improvements in asthma symptoms [33]. Phase III trials investigating tiotropium efficacy and tolerability in the paediatric setting have shown positive results across a range of severities, including symptomatic severe asthma. In the VivaTinA-asthma trial, a 12-week study involving 400 children (aged 6–11 years) with symptomatic severe asthma receiving ICS plus ≥1 controller therapy as maintenance treatment, tiotropium 5 *μ*g add-on improved peak FEV_1(0–3h)_ response at Week 12 versus placebo add-on (adjusted mean difference: 139 mL, 95% confidence interval [CI] 75–203, P<0.001) [34]. Tiotropium has also been evaluated in 392 adolescent patients (aged 12–17 years) with symptomatic severe asthma receiving ICS plus ≥1 controller therapy in the PensieTinA-asthma trial [35]. This 12-week parallel assignment trial did not meet the primary endpoint, with tiotropium 5 *μ*g add-on therapy only numerically improving peak FEV_1(0–3h)_ response versus placebo add-on at Week 12 (90 mL, 95% CI −19 to 198, P=0.104). However, the lower dose of tiotropium 2.5 *μ*g did show nominally significant improvement in peak FEV_1(0–3h)_ response versus placebo add-on at Week 12 (111 mL, 95% CI 2–220, P=0.046). Both the VivaTinA-asthma and PensieTinA-asthma trials investigated the effect of tiotropium add-on treatment on symptoms via the interviewer-administered version of the Asthma Control Questionnaire (ACQ-IA) and the ACQ-7, respectively. Both trials reported no significant difference in responder rate between tiotropium add-on and placebo [34, 35]; however, there was improvement in ACQ-AI or ACQ-7 score in all treatment arms, including the placebo groups, possibly due to improved background medication compliance in the trial setting [36, 37]. This strong placebo effect makes interpretation of these trial results challenging. Importantly, both the PensieTinA-asthma and VivaTinA-asthma trials found that tiotropium add-on therapy was well tolerated, with comparable or lower numbers of patients reporting adverse events compared with placebo.

A recent meta-analysis of the PensieTinA-asthma and VivaTinA-asthma trials, pooling data from 792 paediatric patients, found that peak FEV_1(0–3h)_ response at Week 12 was significantly improved in patients receiving tiotropium add-on versus placebo (tiotropium 5 *μ*g: 117 mL, P=0.0005; tiotropium 2.5 *μ*g: 74 mL, P=0.0273) [38]. Similarly, trough FEV_1_ response was significantly greater with tiotropium 5 *μ*g add-on versus placebo (tiotropium 5 *μ*g: 71 mL, P=0.0395; tiotropium 2.5 *μ*g: 64 mL, P=0.0617). Patients receiving tiotropium add-on versus placebo were found to have significantly greater forced expiratory flow at 25–75% of forced vital capacity (FVC) (FEF_(25–75%)_) response (tiotropium 5 *μ*g: 296 mL/sec, P<0.0001; tiotropium 2.5 *μ*g: 211 mL/sec, P=0.0012) and trough FEV_1_/FVC ratio (tiotropium 5 *μ*g: 1.921%, P=0.0040; tiotropium 2.5 *μ*g: 1.930%, P=0.0038).

Asthma exacerbations and worsening were not primary endpoints in either the PensieTinA- or VivaTinA-Asthma trials, and thus the trials were not powered toward detecting an effect. In particular, the trial lengths of 12 weeks, agreed upon with the regulatory bodies, were insufficient to detect significant effects on exacerbations in the single trials. Nevertheless, a meta-analysis pooling data from the PensieTinA- and VivaTinA-asthma trials has indicated tiotropium may have some activity in reducing asthma worsening in the paediatric severe asthma setting [39]. Time to first asthma worsening in this pooled analysis of 792 patients was significantly increased with tiotropium compared with placebo (tiotropium 2.5 *μ*g: P=0.009; tiotropium 5 *μ*g: P=0.029).

These data underline the efficacy of tiotropium in severe paediatric asthma and, in line with this, tiotropium 5 *μ*g add-on therapy has recently been approved in the EU for use in children aged 6 years and older with symptomatic asthma [21]. Furthermore, the data from these paediatric trials support those from the PrimoTinA-asthma trials in showing that tiotropium is an efficacious therapy for the treatment of severe asthma, with a significant effect on improving lung function across a broad range of ages. However, this conclusion must be applied within the context of the patient populations studied, namely, adult patients with persistent symptoms and reversible airways obstruction despite receiving high-dose ICS/LABA and paediatric patients aged 6–17 years with persistent symptoms and reversible airways obstruction despite receiving high-dose ICS plus additional controller therapies.

## 3. The Role of LAMAs in Personalized Therapy

Stratifying patients for personalized treatment, especially those with severe asthma, is being discussed as the treatment approach of choice (see other reviews in this special issue). This raises the question: should a personalized treatment approach be applied to tiotropium therapy?

To address this question in an adult patient population with severe asthma, Kerstjens and colleagues performed post hoc analyses using pooled data from the PrimoTinA-asthma trials to determine whether baseline characteristics influenced tiotropium efficacy [30]. The analysis focused on the endpoints peak FEV_1(0–3h)_ response and trough FEV_1_ response at Week 24 and time to first asthma exacerbation and first asthma worsening over 48 weeks. None of these endpoints were significantly influenced by any baseline characteristic investigated, including sex, age, body mass index, disease duration, age of asthma onset, or smoking status, thus supporting the efficacy of tiotropium across a broad range of patients with severe asthma.

Inflammation, both allergic and nonallergic, is a significant feature of asthma. Elevated eosinophilic inflammation and elevated IgE levels, as well as the release of cytokines such as interleukin-5 (IL-5) and interleukin-13, define the T2-high asthma phenotype [40]. The T2-high phenotype is used in clinical practice to stratify patients for biological therapies such as anti-IgE and anti-IL-5 antibodies [41]. Casale and colleagues recently investigated whether the efficacy of tiotropium was influenced by the T2 phenotype status [42]. Their post hoc analysis used data from four large Phase III trials: PrimoTinA-asthma (two replicate trials involving 912 adult patients with symptomatic severe asthma where patients were receiving at least ICS/LABA maintenance therapy) and MezzoTinA-asthma (two replicate trials involving 2100 adult patients with symptomatic moderate asthma where patients were receiving at least ICS maintenance therapy). The analysis found that tiotropium improved lung function versus placebo in all trials regardless of baseline phenotype. Analysis of the PrimoTinA-asthma (severe asthma) trials revealed that tiotropium improved peak FEV_1(0–3h)_ by 102 mL (P<0.01) and 148 mL (P<0.001) versus placebo in patients with both high (>430 *μ*g/L) and low (≤430 *μ*g/L) baseline serum IgE, respectively. Trough FEV_1_ was improved by 89 mL (P<0.01) and 127 mL (P<0.001) in patients with both high and low serum IgE levels, respectively. Baseline serum IgE levels also had no significant effect on the risk of exacerbation for tiotropium versus placebo (interaction P=0.17) [30]. The authors reported similar improvements in peak FEV_1(0–3h)_ response and trough FEV_1_ response for all patients, irrespective of whether they were categorized as having allergic asthma by clinician judgement at baseline. Casale and colleagues also modelled the treatment effect of tiotropium over continuous ranges of phenotype biomarkers [42]. Their analysis found improvements in peak FEV_1(0–3h)_ response and trough FEV_1_ response in patients across a broad range of serum IgE levels and blood eosinophil counts at baseline. In addition to lung function improvements, the analysis indicated that asthma symptoms measured by the ACQ-7 score and the risk of asthma worsening were consistently improved with tiotropium therapy in patients with severe asthma across a range of serum IgE levels. This analysis suggests that there is no benefit in determining T2 phenotype status for the selection of patients with severe asthma who will benefit from tiotropium therapy.

A similar post hoc analysis has been conducted using pooled data from clinical trials involving paediatric patients (aged 6–17 years) with moderate or severe asthma receiving placebo or tiotropium add-on therapy [43]. As with Casale et al., modelling of lung function endpoints across a continuum of baseline blood eosinophil counts and serum IgE levels was performed. The study found that peak FEV_1(0–3h)_ response, trough FEV_1_ response, FEV_1_/FVC ratio, FEF_25–75%_ response, and in-clinic trough (evening) peak expiratory flow response improved with tiotropium therapy regardless of eosinophil blood count or IgE serum levels.

These findings in adult and paediatric patients, across a range of baseline characteristics, are perhaps to be expected because, as a bronchodilator, tiotropium should be beneficial in any patient with reversible airway obstruction. However, the results do provide important evidence that tiotropium is efficacious independent of disease subtype, negating the need for patient stratification. As such, tiotropium may be ideally placed as a therapy to be trialled in patients with uncontrolled severe asthma before undergoing phenotyping tests and pursuing personalized biological therapies. An important consideration is that we are unable to determine from current studies the extent to which tiotropium add-on therapy could reduce the number of patients requiring biologic treatment, although such data would be of great interest. However, the evidence presented would imply that a proportion of patients would gain benefit, and that this is irrespective of T2 status and therefore would not require prior phenotyping of patients.

## 4. LAMAs: A Cost-Effective Therapy for Severe Asthma?

An important consideration for biological therapy is cost. These therapies come with a significant economic burden for healthcare providers; for example, the estimated cost for the anti-IgE omalizumab and the anti-IL-5 mepolizumab monoclonal antibodies is $437 and $625 per patient per week, respectively [44, 45]. It is therefore prudent to tailor treatment strategies in such a way that the only patients to receive these expensive biological therapies are those that will benefit from them. As treatment is escalated for uncontrolled asthma, patients should trial each therapy in a systematic manner, as recommended in the 2018 GINA report [5]. As discussed above, tiotropium is an efficacious LAMA for patients with severe asthma irrespective of various phenotype characteristics. Tiotropium is therefore an obvious choice to be trialled during treatment step-up for patients with uncontrolled severe asthma, and the guidelines reflect this [2, 5].

Analysis of the cost-effectiveness of tiotropium in terms of improving asthma control and preventing exacerbations for patients with uncontrolled severe asthma has been conducted in the context of the UK healthcare system [46, 47]. Using 2012 prices, the authors reported the cost of tiotropium per patient per week to be £8.28, with lifetime cost over standard care to be £5389. Guidelines by the UK regulator, the National Institute for Health and Care Excellence, stipulate that an intervention must have a maximum threshold of £30,000 per quality-adjusted life year (QALY) in order to be classed as cost-effective. Analysis of tiotropium benefit revealed the addition of 0.19 QALYs over standard care, giving tiotropium a cost-effectiveness of £28,383 per QALY in the model. The authors therefore concluded this was a cost-effective intervention.

Recently, a study investigated the cost effectiveness of tiotropium in the US healthcare setting in patients with uncontrolled severe asthma [48]. The study used pricing data adjusted to the 2013 US consumer price index, reporting tiotropium cost per patient per week to be $13. The model estimated the lifetime cost of tiotropium therapy to be $3103 more than standard care. Furthermore, the authors reported that tiotropium add-on therapy added 0.09 QALYs over standard care. The cost-effectiveness of tiotropium add-on therapy in the analysis was $34,478 per QALY compared with standard care. The authors concluded that tiotropium was below a willingness-to-pay threshold of $50,000 per QALY and is therefore not a cost-effective treatment. The authors also compared tiotropium with omalizumab therapy in their cost-effectiveness model. Based on a price per patient per week of $437, the estimated lifetime cost of omalizumab compared with standard care or tiotropium was $179,415 and $176,312, respectively. Omalizumab added 0.38 QALYs over standard care and 0.29 QALYs over tiotropium. However, the high cost of omalizumab therapy meant cost-effectiveness was found to be $463,605 per QALY when compared with standard care and $593,643 per QALY when compared with tiotropium. The authors therefore concluded that tiotropium add-on therapy was more cost-effective than omalizumab and a cost-effective option compared with standard treatment.

Together, these studies suggest tiotropium is a relatively inexpensive and cost-effective therapy when stepping up treatment for patients with severe asthma. However, this cost-effectiveness calculation is only applicable in a scenario where the use of tiotropium in patients with severe uncontrolled asthma results in sufficient quality of life improvements such that a step-up to a personalized biologic treatment is negated. Confirmatory studies are required to demonstrate such a biologic-sparing cost-benefit advantage for tiotropium. These would provide a better measure of the cost-effectiveness of tiotropium as a step-up treatment positioned between ICS/LABA and biologics.

## 5. Conclusions

An important aspect of severe asthma management is the use of add-on treatment to gain control of a patient's disease. This process should be stepwise and continuously reassessed, with appropriate therapies trialled to ensure patients receive the optimum treatment level required to control their asthma.

There is a substantial volume of data indicating that the LAMA tiotropium is an efficacious add-on treatment for use in patients whose severe asthma remains uncontrolled despite combination therapy with ICS and additional controller therapies. The evidence shows improvements in lung function measures, as well as an indication of reductions in risk of and time to asthma exacerbation or worsening and symptom reduction. Importantly, post hoc analyses have suggested that tiotropium is broadly efficacious irrespective of asthma phenotype, meaning tiotropium may be utilized without additional characterization of a patient's asthma. Since better daily control and higher lung function act in a protective manner against loss of control/exacerbation in the long run, tiotropium might help to stabilize patients. As such, the GINA 2018 report recommends tiotropium as an add-on therapy to ICS/LABA before stepping up to biologics for patients with uncontrolled asthma.

Tiotropium is cost-effective and substantially less expensive than biological therapies. Hence, it seems that tiotropium is ideally placed as an add-on therapy that can be trialled in patients prior to additional phenotype-guided therapies or increased ICS dose. This is particularly important in children and adolescents, in whom high-dose ICS is linked to impaired growth. There is a need to conduct further studies in this area to confirm that treatment with tiotropium can reduce the need to step up treatment to phenotype-guided therapies and to calculate the possible cost savings associated with this, in patients with severe asthma. Despite this, escalation to phenotype-specific personalized biological therapies may still be required when asthma remains uncontrolled despite active management and comprehensive trialling of add-on therapies.

## Figures and Tables

**Table 1:** Phase III trials investigating tiotropium in adults, adolescents, and children with severe asthma.

**Trial(s)**	**ClinicalTrials.gov number(s)**	**Background treatment**	**Age, years**	**Trial duration, weeks**	**Week of primary endpoint reporting**	**Patients, n**	**Tiotropium 5 ** ***μ*** **g versus placebo** ^**a**^ **, mL**	**Tiotropium 2.5 ** ***μ*** **g versus placebo** ^**a**^ **, mL**
PrimoTinA-asthma [25]	NCT00772538 NCT00776984	High-dose ICS + LABA	18–75	48	24	912	**Peak FEV**_**1(0–3h)**_ ** response: **86, P<0.05 154, P<0.001 **Trough FEV**_**1**_** response: **88, P<0.01 111, P<0.001 **ACQ response: **–0.13, P=0.06 –0.20, P=0.003 **ACQ responder rate:**NR	N/A

PensieTinA-asthma [35]	NCT01277523	High-dose ICS + ≥1 controllers^b^ or Medium-dose ICS + ≥2 controllers^b^	12–17	12	12	392	**Peak FEV**_**1(0–3h)**_ ** response: **90, P=0.104 **Trough FEV**_**1**_** response: **54, P=0.361 **ACQ-7 response: **0.036, P=NR **ACQ-7 responder rate: **NR, P=0.952	**Peak FEV**_**1(0–3h)**_ ** response: **111, P=0.046 **Trough FEV**_**1**_** response: **115, P=0.051 **ACQ-7 response: **0.058, P=NR **ACQ-7 responder rate: **NR, P=0.802

VivaTinA-asthma [34]	NCT01634152	High-dose ICS + ≥1 controllers^b^ or Medium-dose ICS + ≥2 controllers^b^	6–11	12	12	400	**Peak FEV**_**1(0–3h)**_ ** response: **139, P<0.001 **Trough FEV**_**1**_** response: **87, P=0.01 **ACQ-IA response: **–0.08, P=0.32 **ACQ-IA responder rate: **80.8% vs. 76.9%, P=NR	**Peak FEV**_**1(0–3h)**_ ** response: **35, P=0.27 **Trough FEV**_**1**_** response: **18, P=0.59 **ACQ-IA response: **0.02, P=0.80 **ACQ-IA responder rate: **79.4% vs. 76.9%, P=NR

^a^At week of primary endpoint reporting; ^b^e.g., LABA and/or leukotriene receptor antagonist and/or sustained-release theophylline. ACQ-7, seven-question Asthma Control Questionnaire; ACQ-IA, interviewer-administered version of the Asthma Control Questionnaire; FEV_1_, forced expiratory volume in 1s; FEV_1(0–3h)_, FEV_1_ within 3 hours after dose; LABA, long-acting *β*_2_-agonsit; N/A, not applicable; NR, not reported; NS, not significant.
