# Corrigendum to “Managing Severe Asthma: A Role for the Long-Acting Muscarinic Antagonist Tiotropium”

**DOI:** 10.1155/2019/8515804

**Published:** 2019-01-29

**Authors:** Eckard Hamelmann

**Affiliations:** Children's Center Bethel, Ev. Klinikum Bielefeld gGmbH, Grenzweg 10, 33617 Bielefeld, Germany

In the article titled “Managing Severe Asthma: A Role for the Long-Acting Muscarinic Antagonist Tiotropium” [1], there were errors in the description of Figure 1 and Table 1. The last sentence of Figure 1 should be updated from “LABA, long-acting *β*2-agonist” to “IL, interleukin; LABA, long-acting *β*2-agonist; LTRA, leukotriene receptor antagonist; OCS, oral corticosteroid; SABA, short-acting *β*2-agonist.” In addition, the last sentence of Table 1 should be updated from “LABA, long-acting *β*2-agonist; N/A, not applicable; NR, not reported; NS, not significant" to “ICS, inhaled corticosteroid; LABA, long-acting *β*2-agonist; N/A, not applicable; NR, not reported.”
